# Stomatological disorders in older people: An epidemiological study in the brazil southern

**DOI:** 10.4317/medoral.22966

**Published:** 2019-08-18

**Authors:** Eduarda Fattori, Dieni-da Silveira Teixeira, Maria-Antonia-Zancanaro de Figueiredo, Karen Cherubini, Fernanda-Gonçalves Salum

**Affiliations:** 1 Oral Medicine Division, Pontifical Catholic University of Rio Grande do Sul-PUCRS, Porto Alegre, RS, Brazil

## Abstract

**Background:**

The aim of this retrospective, cross-sectional and observational study was to perform a survey of the stomatological conditions of elderly patients seen in a period of 40 years at a Stomatology Service in Southern Brazil.

**Material and Methods:**

A total of 24,347 medical records were reviewed, of which 5,063 belonged to elderly patients aged 60 to 97 years. The stomatological conditions, systemic conditions, and smoking and alcohol drinking habits as well were recorded.

**Results:**

The mean age of the patients was 69.29 years, 67.1% were female and 32.9% were male. Variations of normality accounted for 44.5% of the cases. The most prevalent disorders were fungal infections (26.1%), reactive inflammatory lesions (24.6%), burning mouth syndrome (14.9%), benign neoplasms (12.4%), autoimmune disorders (12.3%), premalignant lesions (10.2%) and malignant epithelial neoplasms (7.2%). Regarding biopsied lesions, squamous cell carcinoma was the most prevalent at 30.2%, followed by hyperplasic lesions (28.2%).

**Conclusions:**

Knowledge of these physiological and pathological conditions in the oral cavity of the older people is essential for early diagnosis and preventive and therapeutic measures when necessary.

** Key words:**Oral mucosa, aged, oral medicine, elderly, oral lesions.

## Introduction

In the last decades there has been a rise in the life expectancy of the population, with a significant increase in the proportion of the older people (1-3), due to improvements in sanitary and housing conditions, advances in medicine, public policies directed at the older people, reduction of infant and child mortality, and greater access to education, among other factors (1,4-5). Oral health has a direct impact on overall health and quality of life. Dental and periodontal changes may affect the nutritional status and physical and mental well-being of elderly individuals (6). In addition, with the advancement of age, the likelihood of lesions appearing in the oral mucosa seems to increase, which is due to systemic diseases, difficulties with hygiene, use of poorly fitting dentures, smoking, alcohol use, hyposalivation and use of medications (7). The oral mucosa, even clinically normal, shows changes due to aging, such as a decrease in the thickness of the epithelium and keratin layer (1). The mucosa becomes thinner, smooth, friable, and subject to injury, and its healing is slower. There is a decrease in taste ability due to the reduction in taste buds, as well as a decrease in the salivary volume, which may hamper chewing, speech and digestion (7).

Studies from different parts of the world suggest that oral mucosal lesions are common in older people (2,8-11). Lynge Pedersen *et al.* (2) conducted a study in the older people in Denmark and found that 75% of subjects had at least one stomatological condition. Lingual varicosities, denture stomatitis, candidiasis, fissured tongue and frictional keratosis were the most prevalent conditions. In a retrospective study by Rivera *et al.* (11), in a Chilean elderly population, fibroma, hemangioma, burning mouth syndrome (BMS) and lichen planus were the most common diseases. Silva *et al.* (10) evaluated four Brazilian referral centers in oral diagnosis, finding inflammatory lesions and neoplasias as the most prevalent changes in patients older than 60 years. Among neoplasias, 57.5% were malignant tumors and 42.5% were benign. The most common malignant tumor was squamous cell carcinoma, and fibroma for the benign tumors.

The aim of the present retrospective study was to determine the prevalence of stomatological conditions in elderly patients seen at a stomatology referral service in southern Brazil over a period of 40 years. We analyzed the clinical diagnoses of lesions and stomatological conditions and the histopathological diagnoses of biopsied oral lesions as well. We investigated associations between gender, systemic diseases/medical conditions, tobacco and alcohol use and oral mucosal lesions in a sample 60–97 years old.

## Material and Methods

The present retrospective cross-sectional and observational study was approved by the Research Ethics Committee of the Pontifical Catholic University of Rio Grande do Sul (PUCRS) under protocol 34599414.9.0000.5336. The sample consisted of patients aged 60 years and over, seen at the Service of Stomatology and Prevention of Oral Maxillofacial Cancer of São Lucas Hospital - PUCRS from 1977 to 2016. This is a Service of reference in oral diagnosis and a team of specialists in Stomatology attended all patients.

A total of 24,347 medical records were analyzed by a single examiner. The reviewer was trained by one of the team’s specialists, and reviewed every diagnosis. Incomplete records for the information investigated were excluded. If a biopsy was performed, the histopathological diagnosis of the lesions was also recorded. The following data of the patients were also recorded: sex, age, and tobacco and alcohol use, in addition to the presence of systemic conditions. Many patients wore dentures, but most of the records did not make note of this, making it impossible to analyze this information. If the reviewer had any queries about diagnosis or other information of the medical records, one specialist of the team was consulted.

To better analyze the diagnosis, we chose to classify them into 13 different groups. The lesions that did not fit in the bellow-mentioned groups were categorized as “others”:

- Local fungal infections (candidiasis in its different clinical forms)

- Reactive inflammatory lesions (fibroepithelial hyperplasia, papillomatous hyperplasia of the palate, gingival hyperplasia, pyogenic granuloma and peripheral giant cell lesion) 

- Variations of normality (varicosities, fissured tongue, geographic tongue, hairy tongue, Fordyce granules, palatine torus, mandibular torus and melanosis)

- Burning mouth syndrome 

- Autoimmune diseases (recurrent aphthous ulceration, lichen planus, lupus erythematosus, Sjögren’s syndrome)

- Premalignant lesions (actinic cheilitis, leukoplakia, erythroplakia and speckled leukoplakia)

- Anemias

- Changes in salivary glands (excluding neoplasms)

- Benign neoplasms 

- Malignant epithelial neoplasms

- Odontogenic cysts and tumors

- Viral infections

- Malignant mesenchymal neoplasms

-Statistical analysis

The data were initially analyzed by descriptive statistics, and the chi-square test was then used to determine the association between the variables studied. A multiple logistic regression model was used, and the variables premalignant lesions, malignant epithelial neoplasms and local fungal infections were adjusted to sex, age, diabetes, depression, smoking and alcohol use. The data collected were tabulated and analyzed using the SPSS program, version 18.0 for Windows. A *p* value ≤ 0.05 was considered significant.

## Results

A total of 5,265 medical records belonged to elderly patients, but 202 ones were excluded because there were no complete information to be analyzed. The sample consisted of 5,063 elderly patients, which totaled 20.79% of the 24,347 medical records reviewed. The individuals’ ages ranged from 60 to 97 years, with a mean of 69.29 (± 7.09) years. Of these patients, 67.1% were female, with a mean age of 69.51 years, and 32.9% were males, with a mean age of 68.84 years.

The distribution of clinical diagnoses and the differences between sexes are presented in the  Table 1. Variations of normality displayed the highest prevalence, representing 44.5% of the cases. Local fungal infections were observed in 26.1% of cases, followed by reactive inflammatory lesions (24.6%), BMS (14.9%), benign neoplasms (12.4%), autoimmune diseases (12.3%), malignant epithelial neoplasms (7.2%), anemias (4.3%), changes in salivary gland (2.7%), odontogenic cysts and tumors (0.9%), viral infections (0.3%) and malignant mesenchymal neoplasms (0.2%). Conditions classified as “others” accounted for 5.7% of cases.

**Table 1:**
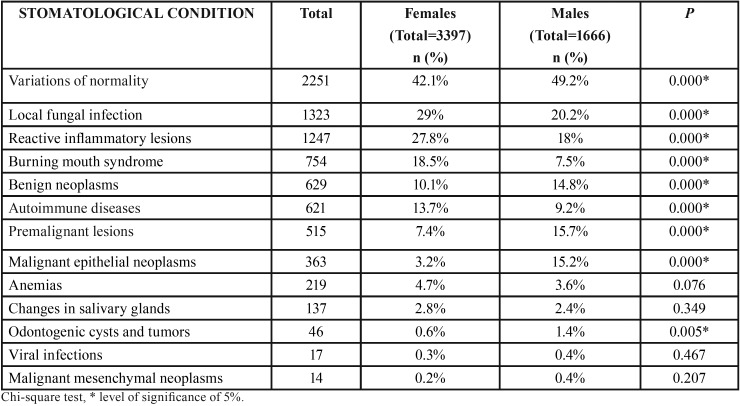
Distribution of stomatological conditions between females and males.

The multiple logistic regression model demonstrate that male had more risk to premalignant lesions (OR 2.27, 95% CI 1.86 – 2.77). It also demonstrated that male, smokers, former-smokers, alcohol users and former-alcohol users had more risk to malignant epithelial neoplasms ( Table 2). Females, diabetic patients, smokers, former-smokers, alcohol users and former- alcohol users had more risk to local fungal infection ( Table 3).

**Table 2:**
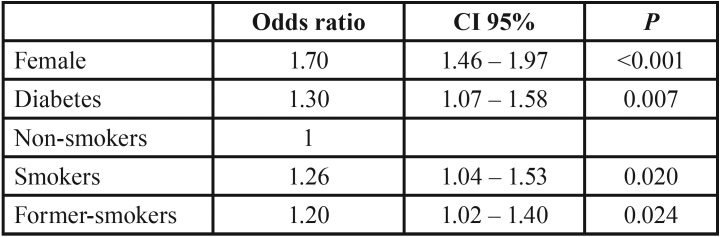
Multiple logistic regression model of factors associated with local fungal infection. The variable was adjusted to sex, age, diabetes, depression, smoking and alcohol use.

**Table 3:**
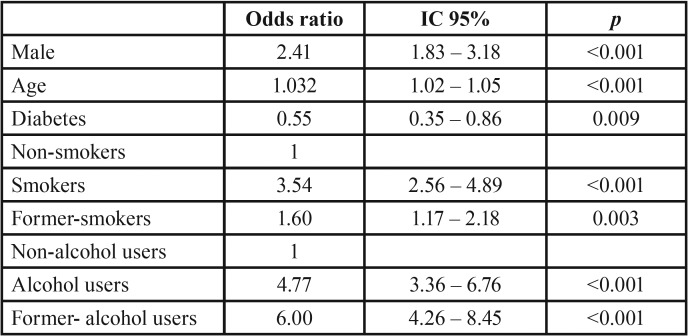
Multiple logistic regression model of factors associated with malignant epithelial neoplasms. The variable was adjusted to sex, age, diabetes, depression, smoking and alcohol use.

The systemic conditions most frequently reported by the patients were hypertension (41.5%), diabetes (11.2%) and depression (5.6%). There was a statistically significant association between depression and BMS (chi-square test, *P*<0.009).

Variations of normality, benign neoplasms, premalignant lesions, malignant epithelial neoplasms, odontogenic cysts and tumors exhibited male predilection (chi-square test, *P*<0.005). On the other hand, local fungal infections, reactive inflammatory lesions, BMS and autoimmune diseases were associated with the female gender (chi-square test, *P*<0.000).

Of the 5063 patients studied, 1048 were subjected to histopathological examination, representing about 20.7% of the sample. The different histopathological diagnoses are presented in  Table 4. The most common histopathological diagnosis was squamous cell carcinoma, followed by inflammatory hyperplasia. Of the cases diagnosed as carcinoma, 230 were male patients and 87 female (chi-square test *P*<0.000). With regard to inflammatory hyperplasia, 241 cases were diagnosed in females and 55 in males (chi-square test, *P*<0.000).

**Table 4:**
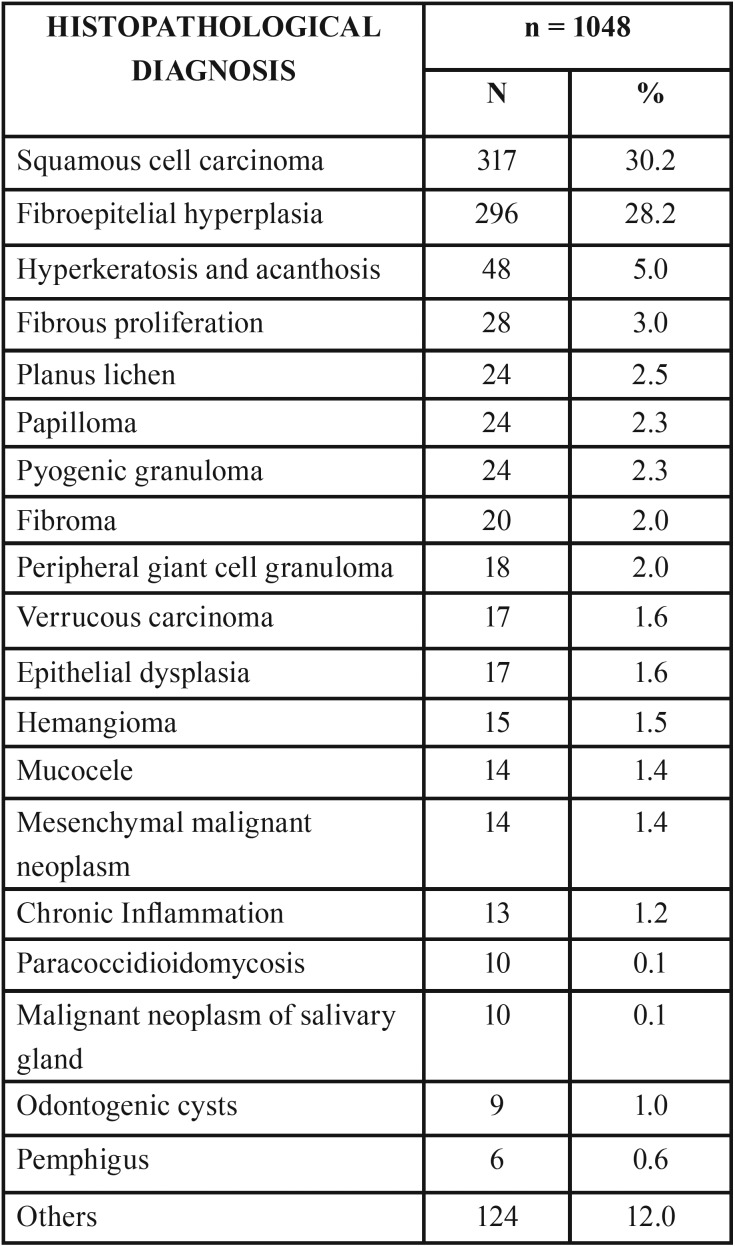
Histopathological diagnosis of biopsied lesions (n=1048) in the sample.

## Discussion

In the present retrospective study, we performed a survey of the stomatological conditions of 5063 elderly patients seen at a Stomatology service in the southern region of Brazil over 40 years. The study was carried out in a database of more than 24,000 medical records, allowing the obtaining of various information regarding those conditions. The clinical and histopathological diagnoses provided in the medical records were analyzed, along with data on smoking, alcohol use and systemic conditions. Almost 70% of the patients in the sample were females. In similar epidemiological studies, Mujica *et al.* (12) and Saintrain *et al.* (13) also observed a higher prevalence of female patients. It should be noted that women seek medical care more frequently. Moreover, in Brazil, life expectancy is higher in females than males, being 78.6 versus 71.3 years old, respectively (5).

To facilitate the presentation of the results, we chose to categorize the clinical diagnoses. The categorization of the lesions was important to observe the general picture of these conditions in the study population. Variations of normality showed the highest prevalence, since in this classification a range of clinical conditions were included. Corroborating this result, Mansour Ghanaei *et al.* (14) observed in an adult Iranian population that the most prevalent oral condition was fissured tongue, followed by Fordyce granules, geographic tongue and melanosis. Shet *et al.* (15) and Lynge Pedersen *et al.* (2) found the lingual varicosities as the most prevalent alteration in the elderly population. The high prevalence of these conditions in the older people may be associated with physiological changes in the aging process. The lower elasticity of vessel walls, for example, is associated with lingual varicosities. The decrease in the thickness of the oral mucosa makes the identification of Fordyce granules more evident. In addition, hyposalivation, common in the older people, is one of the causes of fissured tongue (7).

The most prevalent oral lesion in the sample studied was local fungal infection, represented by cases of erythematous candidiasis, angular cheilitis, pseudomembranous candidiasis, median rhombic glossitis and chronic candidiasis. In the study by Mujica *et al.* (12), the most frequent lesion was denture stomatitis, a form of erythematous candidiasis that occurs on the basis of removable partial or complete dentures. Espinoza *et al.* (16), when evaluating the elderly population in Santiago, Chile, also found that the most frequent oral lesion was candidiasis. This lesion was the second most prevalent in an epidemiological study in the older people conducted by Patil *et al.* (17). This fungal infection is more prevalent in elderly individuals due to hyposalivation, poorly fitting or cleaned dentures, diabetes mellitus, and nutritional deficiencies, among others. In the present study, local fungal infections were associated with diabetes, as this disease alters the immune response of the individual. Bianchi *et al.* (18) found that elderly patients with diabetes had a 4.4-fold higher risk of developing oral candidiasis, reinforcing the results found in our study.

Reactive inflammatory lesions were the third most frequent classification in the present study, demonstrating that this is a significant condition in the elderly population, being mainly related to traumatic factors of low duration and long intensity. Similar results were reported by Espinoza *et al.* (16) and Shet *et al.* (15) in studies in the elderly population. In addition to this, fibroepithelial hyperplasia, which was classified in the group of reactive inflammatory lesions, 

was the second most frequent histopathological diagnosis. Corroborating this data, Dhanuthai *et al.* (9) also observed that fibroepithelial hyperplasia was the second most prevalent oral lesion among biopsies.

BMS was the fourth most frequent classification in our study, representing 14.9% of the diagnoses of this sample. This disease is abstruse and is mainly characterized by symptoms of pain and burning in the oral mucosa without clinical alterations. Rivera *et al.* (11), in a retrospective study in the Chilean elderly population, also found a large number of cases of BMS, being the second most common condition in females. Its etiology is unknown, but studies have related this syndrome to local, systemic and/or psychosocial factors. In our study, a positive association was observed between this disease and depression. Balasubramaniam *et al.* (19) found that one-third of patients with BMS may have underlying psychological diagnosis of depression and anxiety. Bergdahl and Bergdahl (20) also found an association of burning mouth syndrome with depression, corroborating the results of the present study.

The most common histopathological diagnosis was squamous cell carcinoma, followed by fibroepitelial hyperplasia. In three epidemiological studies carried out in referral centers for stomatology and oral pathology in several countries, the most biopsied lesions in the older people were also squamous cell carcinoma and fibroepithelial hyperplasia (8-10). Qannam and Bello (21) performed 231 biopsies in the Saudi elderly, and the most prevalent diagnoses were squamous cell carcinoma and fibroma. These lesions were found in the same proportion, contrasting our findings.

Squamous cell carcinoma is the most prevalent malignant neoplasm of the oral cavity, being strongly associated with tobacco and alcohol use (22). In the present study malignant epithelial neoplasms were associated with these risk factors. Another factor to consider was that smoking and alcohol use were more prevalent in males and, consequently, these lesions were also associated with males, as described in the literature. Another limitation of the present study was the lack of detailed information on severity of the habits of smoking and alcohol consumption, we classified patients as smokers, former-smokers, non-smokers; alcohol users, former-alcohol users or non-alcohol users. In addition, an association of the lesions with the use of partial or total removable prostheses was not possible either.

## Conclusions

Due to the increase in the life expectancy of the population, the number of elderly patients seen at odontological clinics is increasing, making it essential for healthcare professionals to be knowledgeable about the oral conditions of these individuals. In this study, the stomatological conditions of more than 5000 elderly patients were reviewed, with variations of normality, fungal infections, reactive lesions and BMS being the most prevalent. In addition, among the biopsied cases, the most prevalent lesion was squamous cell carcinoma. Knowledge of these oral alterations in the older people is essential for their early diagnosis and establishment of preventive and therapeutic measures when necessary.

## References

[1] Abu Eid R, Sawair F, Landini G, Saku T (2012). Age and the architecture of oral mucosa. Age (Dordr).

[2] Lynge Pedersen AM, Nauntofte B, Smidt D, Torpet LA (2015). Oral mucosal lesions in older people: relation to salivary secretion, systemic diseases and medications. Oral Dis.

[3] Thomson WM, Ma S (2014). An ageing population poses dental challenges. Singapore Dent J.

[4] Romero DE, Leite Ida C, Szwarcwald CL (2005). Healthy life expectancy in Brazil: applying the Sullivan method. Cad Saude Publica.

[5] Szwarcwald CL, Souza Júnior PR, Marques AP, Almeida WD, Montilla DE (2016). Inequalities in healthy life expectancy by Brazilian geographic regions: findings from the National Health Survey, 2013. Int J Equity Health.

[6] Allen F, McKenna G, Mata C, Cronin M, Woods N, O'Mahony D (2010). Gerodontology--how big is the challenge in Ireland?. J Ir Dent Assoc.

[7] Gonsalves WC, Wrightson AS, Henry RG (2008). Common oral conditions in older persons. Am Fam Physician.

[8] Souza S, Alves T, Santos J, Oliveira M (2015). Oral Lesions in Elderly Patients in Referral Centers for Oral Lesions of Bahia. Int Arch Otorhinolaryngol.

[9] Dhanuthai K, Rojanawatsirivej S, Somkotra T, Shin HI, Hong SP, Darling M (2016). Geriatric oral lesions: A multicentric study. Geriatr Gerontol Int.

[10] Silva LP, Leite RB, Sobral APV, Arruda JA, Oliveira LV, Noronha MS (2017). Oral and Maxillofacial Lesions Diagnosed in Older People of a Brazilian Population: A Multicentric Study. J Am Geriatr Soc.

[11] Rivera C, Droguett D, Arenas-Márquez MJ (2017). Oral mucosal lesions in a Chilean elderly population: A retrospective study with a systematic review from thirteen countries. J Clin Exp Dent.

[12] Mujica V, Rivera H, Carrero M (2008). Prevalence of oral soft tissue lesions in an elderly venezuelan population. Med Oral Patol Oral Cir Bucal.

[13] Saintrain MV, Almeida CB, Naruse TM, Gonçalves VP (2013). Oral lesions in elderly patients of a community in Brazilian Northeast. Gerodontology.

[14] Mansour Ghanaei F, Joukar F, Rabiei M, Dadashzadeh A, Kord Valeshabad A (2013). Prevalence of oral mucosal lesions in an adult Iranian population. Iran Red Crescent Med J.

[15] Shet R, Shetty SR, M K, Kumar MN, Yadav RD, S S (2013). A study to evaluate the frequency and association of various mucosal conditions among geriatric patients. J Contemp Dent Pract.

[16] Espinoza I, Rojas R, Aranda W, Gamonal J (2003). Prevalence of oral mucosal lesions in elderly people in Santiago, Chile. J Oral Pathol Med.

[17] Patil S, Doni B, Maheshwari S (2015). Prevalence and distribution of oral mucosal lesions in a geriatric Indian population. Can Geriatr J.

[18] Bianchi CM, Bianchi HA, Tadano T, Paula CR, Hoffmann-Santos HD, Leite DP Jr (2016). Factors related to oral candidiasis in elderly users and non-users of removable dental prostheses. Rev Inst Med Trop Sao Paulo.

[19] Balasubramaniam R, Klasser GD, Delcanho R (2009). Separating oral burning from burning mouth syndrome: unravelling a diagnostic enigma. Aust Dent J.

[20] Bergdahl M, Bergdahl J (1999). Burning mouth syndrome: prevalence and associated factors. J Oral Pathol Med.

[21] Qannam A, Bello IO (2016). The range of diagnoses for oral soft-tissue biopsies of geriatric patients in a Saudi Arabian teaching hospital. Saudi Dent J.

[22] Curado MP, Johnson NW, Kerr AR, Silva DRM, Lanfranchi H, Pereira Dl (2016). Oral and oropharynx cancer in South America: incidence, mortality trends, and gaps in public databases as presented to the Global Oral Cancer Forum. Transl Res Oral Oncol.

